# O-08 - EPIDEMIOLOGY OF ESCHERICHIA COLI BACTERAEMIA IN NHS SCOTLAND: RESULTS FROM A SITUATIONAL ASSESSMENT FOLLOWING NATIONAL EXCEEDANCE OF HEALTHCARE ASSOCIATED INFECTIONS

**DOI:** 10.1093/pubmed/fdag046.008

**Published:** 2026-07-09

**Authors:** Ms Melanie Turner, Marianne Simduwa Alsina, Elaine Glass, Dr Fiona Murdoch, Shona Cairns

**Affiliations:** ARHAI Scotland, NHS Scotland Assure, Public Services Delivery Scotland; ARHAI Scotland, NHS Scotland Assure, Public Services Delivery Scotland; ARHAI Scotland, NHS Scotland Assure, Public Services Delivery Scotland; ARHAI Scotland, NHS Scotland Assure, Public Services Delivery Scotland; ARHAI Scotland, NHS Scotland Assure, Public Services Delivery Scotland

## Abstract

**Background:**

E.coli remains one of the leading causes of bloodstream infections in Scotland. A key national target of the UK 5-year action plan for antimicrobial resistance 2024-2029 is to prevent any increase in Gram-negative bloodstream infections including E.coli bacteraemia (ECB) from 2019-2020 financial year baseline. National surveillance identified a recent significant increase in healthcare-associated ECB incidence across NHS Scotland.

**Objectives:**

To describe the recent incidence and demographic distribution of healthcare-associated ECB infections in NHS Scotland with the aim of identifying potential further work needed to inform local and national interventions.

**Methods:**

All E.coli positive blood culture isolates (defined as no previous isolate in the preceding 14 days) were extracted from the Electronic Communication of Surveillance in Scotland (ECOSS) for April 2016-October 2025. Incidence rates were calculated per 100,000 total occupied bed-days (TOBDs).

**Results:**

A national increase in the rate of healthcare-associated ECB was observed in Q2 2025 (44.0 per 100,000 TOBDs) and Q3 2025 (43.2 per 100,000 TOBDs); the highest rates in NHS Scotland since the start of enhanced surveillance in 2016. This increase was mainly driven by healthcare-associated infections not acquired in hospital. Of these, the most common infection sources reported were urinary tract infections (UTI) (Q2 2025 49.6%; Q3 2025 48.8%) and hepatobiliary (Q2 2025 14.6%; Q3 2025 18.3%). For UTI the highest % case distribution was seen in males ≥65 years (Q2 2025 53.7%; Q3 2025 57.9%), and for hepatobiliary: males ≥65 years in Q2 2025 (59.3%), females ≥65 years in Q3 2025 (40.9%). Additional analyses investigating comorbidities, healthcare usage, and prescribing, will allow greater understanding of potential factors behind the increased incidence.

**Conclusions:**

Healthcare-associated infections not acquired in hospital have driven a recent increase in healthcare-associated infections in NHS Scotland. Linkage with NHS Scotland datasets is currently underway to provide further analyses and more comprehensive understanding of the underlying reasons why.

